# Identification and Characterization of Fluoroquinolone Non-susceptible *Streptococcus pyogenes* Clones Harboring Tetracycline and Macrolide Resistance in Shanghai, China

**DOI:** 10.3389/fmicb.2018.00542

**Published:** 2018-03-23

**Authors:** Yinfang Shen, Jiehao Cai, Mark R. Davies, Chi Zhang, Kun Gao, Dan Qiao, Haoqin Jiang, Weilei Yao, Yuefang Li, Mei Zeng, Mingliang Chen

**Affiliations:** ^1^Department of Infectious Diseases, Children's Hospital of Fudan University, Shanghai, China; ^2^Department of Pediatrics, Jinshan Hospital, Fudan University, Shanghai, China; ^3^Department of Microbiology and Immunology, Peter Doherty Institute for Infection and Immunity, University of Melbourne, Melbourne, VIC, Australia; ^4^Department of Microbiology, Shanghai Municipal Center for Disease Control and Prevention, Shanghai, China; ^5^Department of Clinical Laboratory, Xuhui Dahua Hospital, Shanghai, China; ^6^Department of Clinical Laboratory, Ruijin Hospital (North), Shanghai Jiaotong University School of Medicine, Shanghai, China; ^7^Department of Laboratory Medicine, Shanghai Medical College, Huashan Hospital, Fudan University, Shanghai, China; ^8^Department of Microbiology, Shanghai Institutes of Preventive Medicine, Shanghai, China

**Keywords:** *Streptococcus pyogenes*, fluoroquinolone resistance, multidrug resistance, pulsed-field gel electrophoresis (PFGE), horizontal gene transfer

## Abstract

*Streptococcus pyogenes*, also known as group A *Streptococcus* (GAS), is one of the top 10 infectious causes of death worldwide. Macrolide and tetracycline resistant GAS has emerged as a major health concern in China coinciding with an ongoing scarlet fever epidemic. Furthermore, increasing rates of fluoroquinolone (FQ) non-susceptibility within GAS from geographical regions outside of China has also been reported. Fluoroquinolones are the third most commonly prescribed antibiotic in China and is an therapeutic alternative for multi-drug resistant GAS. The purpose of this study was to investigate the epidemiological and molecular features of GAS fluoroquinolone (FQ) non-susceptibility in Shanghai, China. GAS (*n* = 2,258) recovered between 2011 and 2016 from children and adults were tested for FQ-non-susceptibility. Efflux phenotype and mutations in *parC, parE, gyrA*, and *gyrB* were investigated and genetic relationships were determined by *emm* typing, pulsed-field gel electrophoresis and phylogenetic analysis. The frequency of GAS FQ-non-susceptibility was 1.3% (30/2,258), with the phenotype more prevalent in GAS isolated from adults (14.3%) than from children (1.2%). Eighty percent (24/30) of FQ-non-susceptible isolates were also resistant to both macrolides (*ermB*) and tetracycline (*tetM*) including the GAS sequence types *emm*12, *emm*6, *emm*11, and *emm*1. Genomic fingerprinting analysis of the 30 isolates revealed that non-susceptibility may arise in various genetic backgrounds even within a single *emm* type. No efflux phenotype was observed in FQ non-susceptible isolates, and molecular analysis of the quinolone resistance-determining regions (QRDRs) identified several sequence polymorphisms in ParC and ParE, and none in GyrA and GyrB. Expansion of this analysis to 152 publically available GAS whole genome sequences from Hong Kong predicted 7.9% (12/152) of Hong Kong isolates harbored a S79F ParC mutation, of which 66.7% (8/12) were macrolide and tetracycline resistant. Phylogenetic analysis of the *parC* QRDR sequences suggested the possibility that FQ resistance may be acquired through inter-species lateral gene transfer. This study reports the emergence of macrolide, tetracycline, and fluoroquinolone multidrug-resistant clones across several GAS *emm* types including *emm*1 and *emm*12, warranting continual surveillance given the extensive use of fluoroquinolones in clinical use.

## Introduction

*Streptococcus pyogenes*, ranked as one of the top 10 infectious causes of death worldwide, is responsible for more than 517,000 deaths annually (Carapetis et al., 2005). Also known as group A *Streptococcus* (GAS), it can cause various clinical infections, such as pharyngitis, impetigo, scarlet fever, necrotizing fasciitis, streptococcal toxic shock syndrome, and the immune mediated post-infectious manifestations of acute post-streptococcal glomerulonephritis, acute rheumatic fever and rheumatic heart disease (Walker et al., 2014). Since 2011, an unexpected pediatric scarlet fever epidemic has occurred and sustained in Hong Kong and throughout mainland China, with an incidence of 22–31 cases per 100,000 people (Chen et al., 2012; Lau et al., 2012; Tse et al., 2012; Yang et al., 2013; Davies et al., 2015). Furthermore, an ongoing scarlet fever outbreak has been reported in the United Kingdom since 2013/2014 season (Chalker et al., 2017), with an incidence of 25 cases per 100,000 people.

Penicillin and macrolides are primary antibiotic therapeutics administered for suspected *S. pyogenes* infections (Montes et al., 2010). Although *S. pyogenes* remains susceptible to penicillin, resistance to macrolides is increasing and a major cause for concern in China, with a frequency >93% (Chen et al., 2012; Yang et al., 2013). Fluoroquinolones (FQs) are an attractive alternative when patients are hypersensitive to beta-lactam antibiotics (Montes et al., 2010). In China, consumption of FQs increased by up to 2 × 10^8^ standard units during 2001–2010, becoming the third most consumed antibiotic in the adult population (Van Boeckel et al., 2014). Increasing frequencies of *S. pyogenes* isolates with reduced susceptibility to FQs have been observed in many countries (Reinert et al., 2004; Malhotra-Kumar et al., 2005; Smeesters et al., 2009; Montes et al., 2010; Pires et al., 2010; Van Heirstraeten et al., 2012; Petrelli et al., 2014), yet data on FQ-non-susceptible *S. pyogenes* in China remain scarce despite extremely high resistance to primary interventions such as macrolides.

In Gram-positive bacteria, FQ resistance is mainly mediated through point mutations within the quinolone resistance-determining region (QRDR) of topoisomerase IV ParC and ParE and /or the topoisomerase II DNA gyrase GyrA and GyrB and efflux pump (Hooper, 2002). In *S. pyogenes*, FQ resistance is reported to be conferred by either *parC* or *gyrA* (Yan et al., 2000; Albertí et al., 2005; Montes et al., 2010), and develop in a stepwise manner (Malhotra-Kumar et al., 2009). At first, mutations occurring in *parC* can lead to low-level resistance. Then, additional mutations in *gyrA* can lead to high-level resistance. Mutations in the *parC* and *gyrA* QRDRs can occur not only spontaneously but also by means of horizontal gene transfer from other streptococcal species (Pletz et al., 2006; Duesberg et al., 2008; Pinho et al., 2010).

The aim of this study was to investigate the frequency, mechanism, and epidemiological association of FQ non-susceptibility in *S. pyogenes* during 2011 and 2016 from Shanghai, China.

## Materials and methods

### Isolate database

In order to generate a *S. pyogenes* isolate database representative of the Shanghai population, carriage, and clinical isolates were prospectively collected from different geographical locations in Shanghai between June 2011 to June 2016. A total of 2,258 isolates were collected, of which 2,230 were from children ≤15 years old and 28 from adults. The database including 2,094 clinical isolates and 164 carriage isolates mostly from healthy students in schools. Clinical isolates were primarily from pediatric patients presenting with scarlet fever (*n* = 1,717), tonsillitis (*n* = 355), invasive infections (*n* = 1), or Henoch-Schonlein purpura (*n* = 1). *S. pyogenes* isolates were confirmed by latex-agglutination with the Diagnostic Streptococcal Grouping Kit (Oxoid, Hampshire, UK) and Vitek 2 system (bioMérieux, Marcy l'Etoile, France). All 2,258 isolates were tested for fluoroquinolone resistance.

### Molecular typing and genomic fingerprinting of *S. pyogenes*

All isolates were characterized by *emm* type according to the standard protocol of Centers for Disease Control and Prevention (CDC; http://www.cdc.gov/streplab/protocol-emm-type.html). Pulsed-field gel electrophoresis (PFGE), a method involving chromosomal DNA macrorestriction, was performed to describe the genomic relationships among the GAS population using restriction endonuclease *Sma*I (TaKaRa, Dalian, China). PFGE patterns were analyzed using BioNumerics software package (version 6.5; Applied Maths, Austin, TX, USA) under the unweighted pair group method and an arithmetic averages (UPGMA) clustering algorithm, with settings as 1.0% optimization and 1.5% band tolerance (Carrico et al., 2006) As recommended in Hong Kong scarlet fever outbreak investigation (Tse et al., 2012), isolates with 80% similarity of PFGE bands were assigned to the same cluster. In the same cluster, isolates with indistinguishable PFGE pattern (no different bands) were assigned to the same clone.

### FQs susceptibility test

Minimum inhibitory concentration (MIC) of levofloxacin was determined and interpreted with the broth microdilution method procedure recommended by the Clinical and Laboratory Standards Institute (CLSI) guidelines published in 2015 (Clinical and Laboratory Standards Institute, 2015). According to the breakpoints in CLSI 2015, FQ susceptibility were defined as susceptible, intermediate-resistant, and resistant when the isolates with MIC to levofloxacin as ≤2μg/ml, 4μg/ml, and ≥8μg/ml. *S. pyogenes* FQ non-susceptibility was defined as intermediate-resistant and resistant isolates. Efflux pump activity in the FQ non-susceptible isolates was investigated by the agar dilution method according to CLSI 2015 guidelines, comparing the MICs to levofloxacin and ciprofloxacin in the presence or absence of 30 μg/ml reserpine (Sigma-Aldrich), an efflux pump blocker (Jones et al., 2003). When a FQ-non-susceptible isolate showed a four-fold or greater decrease in the MIC to levofloxacin or ciprofloxacin, the efflux phenotype was deemed to be positive, which supported the involvement of the efflux mechanism (Jones et al., 2003).

### Sequencing of quinolone resistance-associated genes

The QRDR sequences of genes encoding the topoisomerase IV(*parC* and *parE*) and the gyrase (*gyrA* and *gyrB*) were amplified and sequenced as previously described (Jones et al., 2003). Sequences of *parC* (accession number AF220946: position 64–561), *parE* (accession number AE004092: 752342–754294), *gyrA* (accession number AF220945: 79–540) and *gyrB* (accession number AE004092: 581675–583627) from the quinolone-susceptible *S. pyogenes* strain SF370 (ATCC 700294) were defined as reference alleles (Malhotra-Kumar et al., 2005). The genome sequences of *S. pyogenes* isolates from Hong Kong and mainland China and the QRDR sequence of *parC* from 2 *S. pneumonia*, 7 *S. agalactiae*, 21 *S. dysgalactiae*, 1 *S. canis*, 1 *S. iniae*, 1 *S. porcinus*, 2 *S. equi*, and 1 *S. difficilis* isolates were retrieved from GenBank with the accession number listed in Tables S1, S2, respectively. The Lasergene software package (version 7.1; DNASTAR, Wisconsin, USA) was used to analyze nucleotide sequences and the deduced amino acid sequences. MEGA 5 (http://www.megasoftware.net/) was used to generate multi-sequence alignments and Neighbor-joining phylogenetic analysis. Clades were determined with the bootstrap values >75% in the bootstrap test with 1,000 replicates. The evolutionary distances were computed by the Kimura two-parameter method and are in the units of the number of base substitutions per site.

### Detection of other resistance-associated genes

Genes associated with macrolide resistance (*ermB, ermA*, and *mef*) and tetracycline resistance (*tetM* and *tetO*) were screened by PCR using primers and reaction parameters as previously described (Pérez-Trallero et al., 2007).

### Statistical analysis

Statistical analysis was performed using SPSS (version 20.0; IBM). Fisher's exact test was used for the comparison of FQ resistance frequency between isolates from children and adults. Statistical significance was assessed at *P* < 0.05.

### Accession numbers

The sequences of the *parC* alleles defined in this study were submitted to GenBank under accession numbers MF278797 to MF278806, while the *parE* QRDR sequences of the 30 FQ-non-susceptible isolates as MG894399 to MG894428.

### Ethical aspects

All GAS isolates characterized in this study were collected from GAS infection cases and their close contacts, as part of routine clinical management of patients and Shanghai surveillance system for scarlet fever, according to guidelines of infectious diseases used in China nationwide. Informed consent from patients and close contacts was approved by Shanghai Municipal Center for Disease Control and Prevention ethical review committee (No: 2016-4) (Chen et al., 2017).

## Results

### FQs susceptibility of *S. pyogenes*

**Figure 1:**
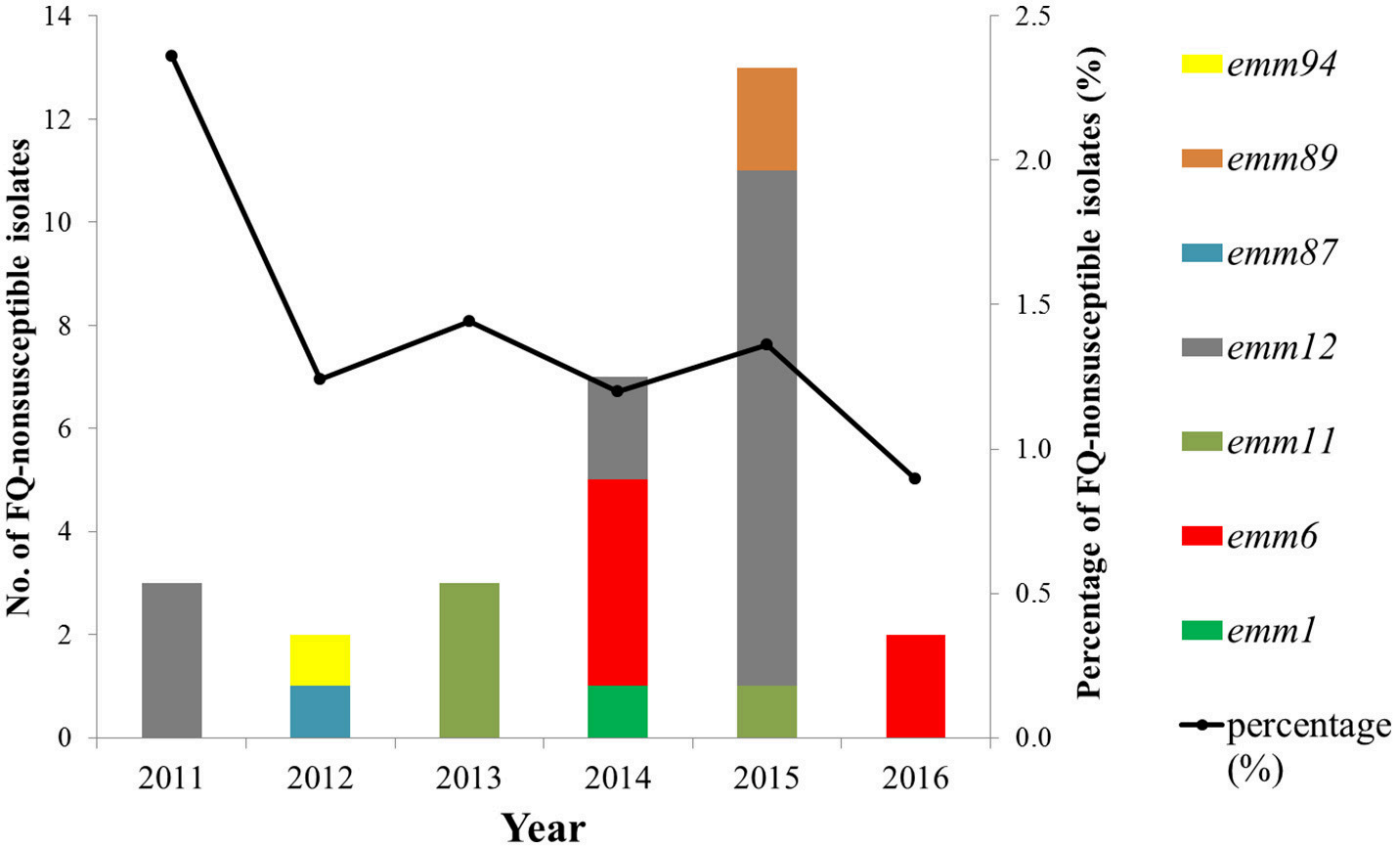
Frequency and *emm*-type distribution of fluoroquinolone non-susceptibility in *S. pyogenes* strains isolated between 2011 to 2016 from Shanghai, China.

A total of 2,258 *S. pyogenes* isolates were collected during 2011–2016, including 2,230 isolates from children ≤15 years old and 28 from adults. There were 30 (1.3%) FQ-non-susceptible isolates; 15 resistant (MIC range 8–16μg/ml) and 15 intermediate-resistant (MIC = 4μg/ml) isolates. The 30 isolates were all from clinical patients. Frequencies of FQ-non-susceptible isolates were different between children and adults (1.2 and 14.3%, respectively; *P* < 0.05). Among the 1,717 isolates from scarlet fever patients, 24 (1.4%) were non-susceptible to FQs. The FQ-non-susceptible isolates were discovered every year during 2011–2016, with the frequency fluctuating between 2.4% in 2011 and 0.9% in 2016 (Figure 1). Alarmingly, twenty-four (80%) of the 30 FQ-non-susceptible isolates were also resistant to erythromycin (>128μg/ml), clindamycin (>128μg/ml), and tetracycline (8-32μg/ml) (Table 1), and were identified to carry the resistance-associated genes *ermB* and *tetM*, while another isolate was resistant to only FQs and tetracycline (32μg/ml), possessing *tetM*.

**Table 1:** Antimicrobial susceptibility of isolates with different *emm* types.

***emm* type**	**Total no. of isolates (%)**	**No. of FQ- non-susceptible isolates (%)^a^**	**No. of isolates resistant to macrolides, tetracycline, and FQ (%)^b^**	**Mutations in FQ-non-susceptible isolates (n)**				**MIC to levofloxacin (μg/ml)^c^**
				**ParC**	**ParE**	**GyrA**	**GyrB**	
*emm*1	816 (36.1)	1 (0.1)	1 (0.1)	S79F (1)	no	no	no	4
*emm*6	6	6 (100)	6 (100)	S79A (6)	A378T (5)	no	no	4–8
*emm*11	4	4 (100)	4 (100)	S79F (4)	S402L (3)	no	no	4–16
*emm*12	1362 (60.3)	15 (1.1)	9 (0.7)	S79F (6), S79Y (8), D83V (1), A121V (15)	no	no	no	4–16
*emm*87	1	1 (100)	1 (100)	S79F (1)	E360D (1), V377L (1), H380N (1)	no	no	4
*emm*89	15	2 (13.3)	2 (13.3)	D78A (1), D91N (2), S140P (2)	E360D (2), D438N (1), D493N (2)	no	no	4–8
*emm*94	1	1 (100)	1 (100)	D83V (1)	no	no	no	4
Others^d^	53	0	0	no	no	no	no	≤ 0.25–2
Total	2258	30 (1.3)	24 (1.1)	D78A (1), S79A (6), S79F (12), S79Y (8), D83V (2), D91N (2), A121V (15), S140P (2)	E360D (3), V377L (1), A378T (5), H380N (1), S402L (3), D438N (1), D493N (2)	no	no	≤ 0.25–16

a *FQ, fluoroquinolone*.

b *Resistance to macrolides and tetracycline in all tested isolates was associated with ermB and tetM gene carriage, respectively*.

c *MIC, minimum inhibitory concentration*.

d *Other emm type isolates included 2x emm3, 3x emm4, 12x emm5, 10x emm22, 10x emm75, 1x emm103, 13x emm170, 1x emm203, and 1x emm227 isolates*.

### Molecular epidemiology of FQ-non-susceptible *S. pyogenes*

Molecular typing of the 2,258 isolates identified 16 *emm* types, with *emm*12 and *emm*1 isolates constituting 60.3 and 36.1% of the isolates respectively (Table 1). The thirty FQ-non-susceptible isolates were assigned to seven *emm* types (Table 1), including *emm*12 (1.1%, 15/1,362), *emm*6 (100%, 6/6), *emm*11 (100%, 4/4), *emm*89 (13.3%, 2/15), *emm*1 (0.1%, 1/712), *emm*87 (100%, 1/1), and *emm*94 (100%, 1/1). With the exception of 6 *emm*12 isolates, all FQ-non-susceptible *emm* types were resistant to both macrolides and tetracycline (Table 1).

**Figure 2:**
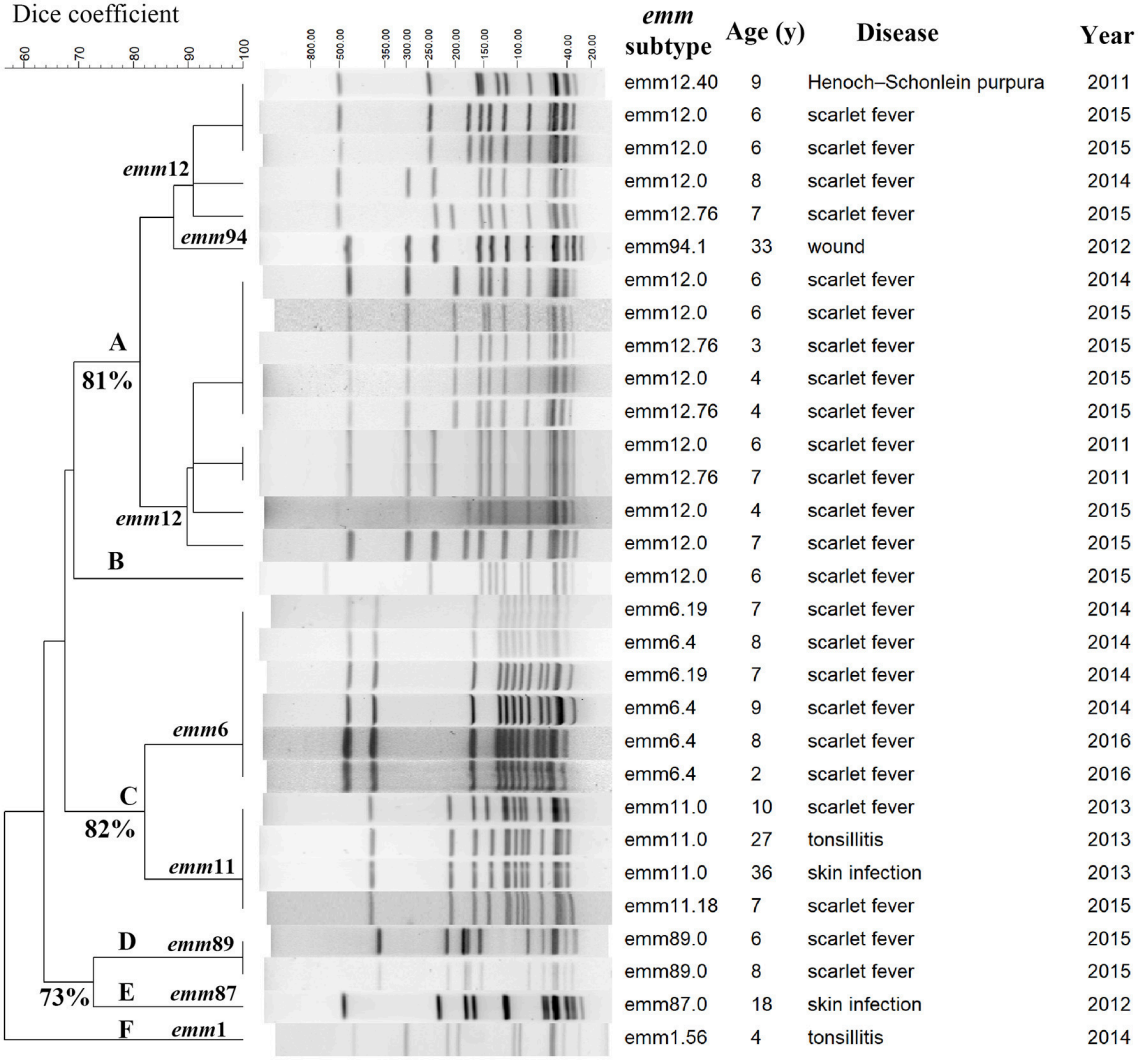
PFGE patterns of 30 fluoroquinolone-non-susceptible *S. pyogenes* strains isolated between 2011 to 2016 in Shanghai, China. Clusters A-F were assigned according to 80% similarity, and, isolates with indistinguishable pulsed-field gel electrophoresis (PFGE) pattern (no different bands) were assigned to the same clone. The numbers of similarity are labeled next to the branches.

To examine the genomic relationships of the FQ-resistant population, genome fingerprinting of the 30 FQ-non-susceptible isolates by PFGE was performed. Six clusters (A-F) were observed (Figure 2). The *emm*6, *emm*11, and *emm*89 isolates were clonal as defined by an identical PFGE pattern whereas the *emm*12 family clustered into multiple subtypes.

### Characterization of FQ resistance mechanisms

To examine the genetic basis of FQ-non-susceptibility, the *parC, parE, gyrA*, and *gyrB* sequences were sequenced and analyzed. Of the 30 FQ-non-susceptible isolates, all harbored at least one amino acid alteration in ParC, 11 (36.7%) harbored ParE mutations, and none had mutations in GyrA and GyrB QRDRs (Table 2). In ParC, alteration at the amino acid site 79 (Ser) was found in 26 (86.7%) FQ-non-susceptible isolates, including S79A (*emm*6), S79F (*emm*1, *emm*11, *emm*12, and *emm*87), and S79Y (*emm*12). The mutation A121V was found in *emm*12 isolates irrespective of the FQ susceptibility. Less common mutations included D78A (1), D91N (2), S140P (2), and a novel mutation, D83V (2). A total of 9 *parC* nucleotide alleles were found in the 30 FQ-non-susceptible isolates, designated as SHparC1- SHparC9 (Table 2). With the exception of SHparC6 that was represented by isolates of *emm*1 and *emm*87, each *parC* allele was represented by isolates of the same *emm* type. In ParE QRDRs, mutations of 7 amino acid sites were identified in 11 FQ-non-susceptible isolates (Table 2), including E360D (3), V377L (1), A378T (5), H380N (1), S402L (3), D438N (1), and D493N (2). To further expand on these findings, we tested the efflux phenotype on all 30 FQ-non-susceptible isolates. None of the FQ-non-susceptible isolates showed a four-fold or greater decrease in the MIC to levofloxacin or ciprofloxacin after adding reserpine (30 μg/ml) indicating that the FQ tolerance could not be explained by the efflux pump mechanism.

**Table 2:** Nucleotide changes identified in the quinolone resistance-determining regions of different *parC* alleles.

***parC* allele**	**MIC to levofloxacin (μg/ml)^a^**	**No. of isolates**	***emm* subtype**	**ParC mutation**	**References to ParC with the same mutation pattern**
SHparC1	4–8	8	12.0 (6), 12.76 (2)	S79Y (TCC→ TAC) A121V^b^ (GCT→ GTT)	Malhotra-Kumar et al., 2005
SHparC2	4–16	6	12.0 (3), 12.40 (1), 12.76 (2)	S79F (TCC→ TTC) A121V (GCT→ GTT)	Richter et al., 2003; Rivera et al., 2005; Lin et al., 2015
SHparC3	4	1	12.0 (1)	D83V (GAT→ GTT) A121V (GCT→ GTT)	This study
SHparC4	4–16	4	11.0 (3), 11.18 (1)	S79F (TCC→ TTC)	Malhotra-Kumar et al., 2005; Orscheln et al., 2005; Biedenbach et al., 2006; Pletz et al., 2006; Montes et al., 2010; Arai et al., 2011
SHparC5	4	1	94.1 (1)	D83V (GAT→ GTT)	This study
SHparC6	4	2	1.56 (1), 87.0 (1)	S79F (TCC→ TTC)	Wajima et al., 2008
SHparC7	4–8	6	6.19 (2), 6.4 (4)	S79A (TCC→ GCC)	Albertí et al., 2005; Malhotra-Kumar et al., 2005, 2009; Orscheln et al., 2005; Powis et al., 2005; Rivera et al., 2005; Yan et al., 2008; Smeesters et al., 2009; Montes et al., 2010; Van Heirstraeten et al., 2012
SHparC8	4	1	89.0 (1)	D91N (GAT→ AAT), S140P (TCT→ CCC)	Malhotra-Kumar et al., 2005
SHparC9	8	1	89.0 (1)	D78A (GAT→ GCT), D91N (GAT→ AAT), S140P (TCT→ CCC)	This study
SHparC10^c^	ND^d^	1	1.0 (1)	D83G (GAT→ GGT)	Van Heirstraeten et al., 2012; Lin et al., 2015

a *MIC, minimum inhibitory concentration*.

b *A121V was also found in fluoroquinolone-susceptible isolates*.

c *Sequence was extracted from the genome of HLJGAS2022*.

d *ND, not determined*.

### Conservation of ParC mutations within whole genome sequences from wider chinese clinical isolates

One hundred and seventy *S. pyogenes* genome sequences of isolates from Hong Kong (152) and mainland China (18) are publically available, including 136 *emm*12 and 34 *emm*1 strains isolated between 2004 to 2012. Examination of the QRDR genes identified 13 (7.6%) isolates with mutations in ParC QRDR, including 12 *emm*12 from Hong Kong belonging to three different evolutionary lineages and 1 *emm*1 isolates from mainland China (Heilongjiang Province), and no mutations within ParE, GyrA, and GyrB. The most common ParC mutation was S79F in ParC (SHparC2) with one *emm*1 isolate harboring alteration D83G in ParC, which was designated as a new *parC* allele: SHparC10.

### Evolution of *parC* mutations within streptococcaceae

**Figure 3:**
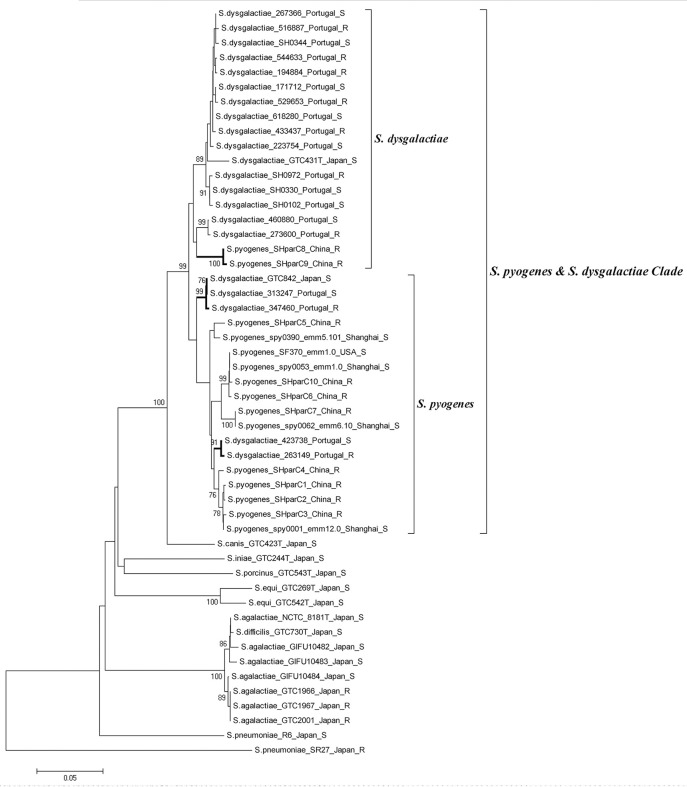
Phylogenetic analysis of *parC* quinolone-resistance-determining region from different streptococcal species. Strains are shown as; species, strain number or *parC* allele; (*emm* type); country or district; and fluoroquinolone susceptibility. All fluoroquinolone-non-susceptible isolates were labeled with “R.” Phylogenetic analysis was conducted in MEGA 5 using Neighbor-joining. Bootstrap values are indicated when support is >75% (from 1,000 replicates). The tree is drawn to scale, with the branch lengths being in the same units as those of the evolutionary distances used to infer the phylogenetic tree. The evolutionary distances were computed by the Kimura two-parameter method and are in the units of the number of base substitutions per site.

Phylogenetic analysis involving 51 QRDR *parC* sequences from *S. pyogenes, S. pneumonia, S. agalactiae, S. dysgalactiae, S. canis, S. iniae, S. porcinus, S. equi*, and *S. difficilis* was performed to examine evidence of lateral gene transfer and FQ resistance. All *S. pyogenes* and *S. dysgalactiae* isolates clustered together into a single clade (Clade *S. pyogenes* & *S. dysgalactiae*, Figure 3). The clade can be further divided into two sub-branches: the *S. pyogenes* sub-branch and the *S. dysgalactiae* sub-branch. Most of the *S. pyogenes parC* alleles (8/10) defined in this study were grouped into *S. pyogenes* sub-branch, while alleles SHparC8 and SHparC9 were found in the *S. dysgalactiae* sub-branch. Five *parC* sequences of *S. dysgalactiae*, including FQ-susceptible and FQ-non-susceptible isolates, were included in the *S. pyogenes* sub-branch.

## Discussion

This study is the first report that investigates and characterizes FQ-non-susceptibility within *S. pyogenes* from mainland China during 2011–2016, the period overlapping an ongoing multidrug resistant GAS scarlet fever epidemic in China (Tse et al., 2012; Yang et al., 2013; Davies et al., 2015). We identified FQ non-susceptibility to be 1.3% in 2,258 GAS isolates from Shanghai, China, including within *emm*12 and *emm*1 scarlet fever clones resistant to macrolides and tetracycline. The appearance of these clones has further narrowed the choices of treatment for GAS infections. We observed a significant difference in FQ resistance frequencies in GAS isolated from adults (14.3%) compared to children (1.2%). This may in part be attributable to clinical practice where administration of fluoroquinolones is largely restricted to adults due to cartilage toxicity observed in children (Stahlmann et al., 1990).

Since 2006, very high frequencies (>90%) of resistance to macrolide and tetracycline in GAS isolates have been reported in mainland China (Jing et al., 2006; Chen et al., 2012; Yang et al., 2013). Coincidently, fluoroquinolone consumption has increased over the last two decades and is currently the third most consumed antibiotic in China (Van Boeckel et al., 2014). Our study identified that 80% of FQ-non-susceptible isolates were also resistant to both macrolides (*ermB*) and tetracycline (*tetM*). In previous GAS studies, only three isolates harboring this multidrug resistance profile have been reported in a Belgian study (Malhotra-Kumar et al., 2005). With the emergence of antimicrobial resistance in respiratory pathogens intrinsically linked with antibiotic consumption (Goossens et al., 2005), the appearance of multidrug resistant clones through selective pressure is highly probable.

Compared with the prevalence of GAS FQ-non-susceptibility in Taiwan (11.1% in 2005–2012), Japan (14.1% in 2010–2012), Belgium (11.4% in 2007–2010), Spain (13% in 2005–2007), and USA (9% in 2003) (Yan et al., 2008; Montes et al., 2010; Van Heirstraeten et al., 2012; Wajima et al., 2013; Lin et al., 2015), the overall prevalence of FQ-non-susceptible *S. pyogenes* in Shanghai (1.3%) and Beijing (3.4%) (Yang et al., 2013) are relatively low. This may be attributed to the limited administration of FQ to pediatric patients in China, which constitute most of isolates in this study (98.8%) and all the isolates in the Beijing study. Correspondingly, low frequencies of FQ resistance was found in the most frequently isolated GAS *emm* type in this study, *emm*12 (1.1%). The frequencies of FQ resistance in *emm*12 isolates varies in different countries, from 0 (0/45) in Spain to 5% (1/20) in Japan, and to 50% (33/66) in Taiwan (Montes et al., 2010; Wajima et al., 2013; Lin et al., 2015). Based on whole genome analysis of scarlet fever isolates in Hong Kong (Davies et al., 2015), we estimate the frequency of *emm*12 FQ resistance in Hong Kong was 9.0% (12/134), of which 66.7% (8/12) were also resistant to macrolide and tetracycline possessing the corresponding molecular markers *ermB* and *tetM* (Table S1); however, validation of these findings through FQ susceptibility testing is required to confirm this genetic observation. The emergence of FQ resistance within ongoing scarlet fever epidemics in Hong Kong and mainland China (Luk et al., 2012; Yang et al., 2013) calls for more precaution when prescribing fluoroquinolones for suspected GAS infections.

In this study, FQ-non-susceptible *emm*6 isolates were discovered in China for the first time. Orscheln et al. proposed that FQ resistance is an intrinsic property of the *emm*6 lineage, with mutations in ParC driving FQs resistance long before the introduction of FQ antibiotics (Orscheln et al., 2005). The global dissemination of *emm*6 strains was suggested to be the main reason for the increased proportion of FQ-non-susceptible *S. pyogenes* in Belgium and Spain (Montes et al., 2010; Van Heirstraeten et al., 2012). All six *emm*6 isolates identified in this study were a single FQ-non-susceptible clone on the basis of PFGE, and harbored the same ParC QRDR S79A mutation (*parC* allele of SHparC7) with those *emm*6 isolates from USA and Spain (Orscheln et al., 2005; Rivera et al., 2005). In this study, the *emm*6 clone was also resistant to erythromycin, clindamycin, and tetracycline. This observation is mirrored for the global *emm*11 clone (Wajima et al., 2008, 2013; Van Heirstraeten et al., 2012) with all four *emm*11 GAS isolated in this study representing a single FQ-non-susceptible clone harboring an S79F ParC mutation and the macrolide and tetracycline resistance markers. Collectively, monitoring for the global dissemination of these multidrug resistant GAS clones is warranted.

All FQ-non-susceptible *S. pyogenes* in this study showed a low-level resistance (MIC range, 4-16 μg/ml) and molecular analyses of the classic FQ-non-susceptibility genetic markers suggests that FQ-non-susceptibility in China is primarily linked to ParC QRDR mutations, for they all harbored ParC mutation(s), occasionally with ParE mutation(s), and none with GyrA and GyrB mutations or involvement of efflux pump, in addition, isolates with ParE mutation(s) showed similar MIC values to levofloxacin and ciprofloxacin compared to those only possessing ParC mutation(s). This supports the stepwise theory of FQ resistance: without GyrA mutation, where mutations in ParC only confer a low-level resistance (Billal et al., 2007). The most common alterations occurred in the amino acid site Ser79, a potential site to bind FQs (Laponogov et al., 2007), which is supported by findings from American and European countries (Orscheln et al., 2005; Smeesters et al., 2009). Polymorphisms of *parC* QRDR were found in FQ-non-susceptible isolates, and they were mostly linked with *emm* types in this study. This suggested most of the mutations might occur spontaneously within the strains' evolutionary process, which was validated by the phylogenetic analysis of the *parC* QRDR however two *parC* alleles may have arisen through horizontal gene transfer from other streptococcal species such as *S. dysgalactiae*. Some *parC* sequence of FQ-susceptible and FQ-non-susceptible *S. dysgalactiae* were also grouped into the *S. pyogenes* sub-branch in this study, which provided more evidence for a global gene pool shared by *S. dysgalactiae* and *S. pyogenes* (Pinho et al., 2010). A number of ParE mutations were identified in this study, including three mutations of E360D, V377L, and H380N concurrently present in a *emm*87 isolate and an A378T mutation identified only in *emm*6 isolates as documented previously (Malhotra-Kumar et al., 2005; Lin et al., 2015), while S402L mutation was only found in *emm*11 isolates, and mutations of D438N and D493N were only identified in *emm*89 isolates, which were first reported in this study. These phenomena suggest most of the ParE mutations might be evolutionary linked with *emm* type rather than drivers of FQ resistance.

One limitation of this study is the small sample size of isolates from adults, which might lead to a possible bias when comparing the FQ-non-susceptibility frequency between isolates from children and adults, but the appearance of adult FQ-non-susceptible isolates in different years (2012 and 2013) supported, to some extent, the validation of the high frequency in adults. The lack of adult isolates can largely be attributed to the fact that only scarlet fever, which is primarily a disease of children, is the only notifiable disease among all the GAS infections in China (Yang et al., 2013). Another possible attribution might be that the morbidity of GAS infections is low in adults; however, more data on adult GAS infections in China is required to support this hypothesis (Chen et al., 2017). Our finding of a higher rate of FQ-non-susceptibility in isolates from adults (14.3%) is alarming and warrants further investigation at a population level.

In conclusion, the emergence of macrolide, tetracycline and fluoroquinolone- non-susceptible *S. pyogenes* in Shanghai, China during the period of scarlet fever epidemics was revealed in this study. Although FQ resistance was infrequent in mainland China, the presence of macrolide, tetracycline and fluoroquinolone multidrug-resistant clones across multiple *S. pyogenes emm* sequence types is alarming and the spread of these isolates should be monitored globally.

## Author contributions

MC and MZ: conceived and designed the experiments; YS, JC, and CZ: performed all experiments; YS, JC, and MD: analyzed the data; YS, KG, DQ, HJ, WY, and YL: collected clinical specimens; MC and MZ: supervised the study and wrote the paper; MD: revised the paper. All authors have read and approved the final manuscript.

### Conflict of interest statement

The authors declare that the research was conducted in the absence of any commercial or financial relationships that could be construed as a potential conflict of interest.

## References

[B1] Albertí S. Cortés G. García-Rey C. Rubio C. Baquero F. García-Rodríguez J. A.. (2005). *Streptococcus pyogenes* pharyngeal isolates with reduced susceptibility to ciprofloxacin in Spain: mechanisms of resistance and clonal diversity. Antimicrob. Agents Chemother. 49, 418–420. 10.1128/AAC.49.1.418-420.2005 15616324 PMC538864

[B2] Arai K. Hirakata Y. Yano H. Kanamori H. Endo S. Hirotani A.. (2011). Emergence of fluoroquinolone-resistant *Streptococcus pyogenes* in Japan by a point mutation leading to a new amino acid substitution. J. Antimicrob. Chemother. 66, 494–498. 10.1093/jac/dkq477 21172783

[B3] Biedenbach D. J. Toleman M. A. Walsh T. R. Jones R. N. (2006). Characterization of fluoroquinolone-resistant beta-hemolytic *Streptococcus* spp. isolated in North America and Europe including the first report of fluoroquinolone-resistant *Streptococcus dysgalactiae* subspecies *equisimilis*: report from the SENTRY Antimicrobial Surveillance Program (1997–2004). Diagn. Microbiol. Infect. Dis. 55, 119–127. 10.1016/j.diagmicrobio.2005.12.006 16530373

[B4] Billal D. S. Fedorko D. P. Yan S. S. Hotomi M. Fujihara K. Nelson N.. (2007). *In vitro* induction and selection of fluoroquinolone-resistant mutants of *Streptococcus pyogenes* strains with multiple emm types. J. Antimicrob. Chemother. 59, 28–34. 10.1093/jac/dkl428 17065188

[B5] Carapetis J. R. Steer A. C. Mulholland E. K. Weber M. (2005). The global burden of group A streptococcal diseases. Lancet Infect. Dis. 5, 685–694. 10.1016/S1473-3099(05)70267-X 16253886

[B6] Carrico J. A. Silva-Costa C. Melo-Cristino J. Pinto F. R. de Lencastre H. Almeida J. S. (2006). Illustration of a common framework for relating multiple typing methods by application to macrolide-resistant *Streptococcus pyogenes*. J. Clin. Microbiol. 244, 2524–2532. 10.1128/JCM.02536-05 PMC1489512 16825375

[B7] Chalker V. Jironkin A. Coelho J. Al-Shahib A. Platt S. Kapatai G.. (2017). Genome analysis following a national increase in Scarlet Fever in England 2014. BMC Genomics 18:224. 10.1186/s12864-017-3603-z 28283023 PMC5345146

[B8] Chen M. Wang W. Tu L. Zheng Y. Pan H. Wang G.. (2017). An *emm*5 Group A Streptococcal outbreak among workers in a factory manufacturing telephone accessories. Front. Microbiol. 8:1156. 10.3389/fmicb.2017.01156 28680421 PMC5478724

[B9] Chen M. Yao W. Wang X. Li Y. Wang G. Zhang X.. (2012). Outbreak of scarlet fever associated with *emm*12 Type group A *Streptococcus* in 2011 in Shanghai, China. Pediatr. Infect. Dis. J. 31, e158–e162. 10.1097/INF.0b013e31825874f3 22531238

[B10] Clinical and Laboratory Standards Institute (2015). Performance Standards for Antimicrobial Susceptibility Testing: Twenty-Fifth Informational Supplement, CLSI Document M100-S25. (Wayne, PA: CLSI).

[B11] Davies M. R. Holden M. T. Coupland P. Chen J. H. Venturini C. Barnett T. C.. (2015). Emergence of scarlet fever *Streptococcus pyogenes emm*12 clones in Hong Kong is associated with toxin acquisition and multidrug resistance. Nat. Genet. 47, 84–87. 10.1038/ng.3147 25401300

[B12] Duesberg C. B. Malhotra-Kumar S. Goossens H. McGee L. Klugman K. P. Welte T.. (2008). Interspecies recombination occurs frequently in quinolone resistance-determining regions of clinical isolates of *Streptococcus pyogenes*. Antimicrob. Agents Chemother. 52, 4191–4193. 10.1128/AAC.00518-08 18765693 PMC2573148

[B13] Goossens H. Ferech M. Vander Stichele R. Elseviers M. Group E. P. (2005). Outpatient antibiotic use in Europe and association with resistance: a cross-national database study. Lancet 365, 579–587. 10.1016/S0140-6736(05)70799-6 15708101

[B14] Hooper D. C. (2002). Fluoroquinolone resistance among Gram-positive cocci. Lancet Infect. Dis. 2, 530–538. 10.1016/S1473-3099(02)00369-9 12206969

[B15] Jing H. B. Ning B. A. Hao H. J. Zheng Y. L. Chang D. Jiang W.. (2006). Epidemiological analysis of group A streptococci recovered from patients in China. J. Med. Microbiol. 55(Pt 8), 1101–1107. 10.1099/jmm.0.46243-0 16849731

[B16] Jones H. E. Brenwald N. P. Owen K. A. Gill M. J. (2003). A multidrug efflux phenotype mutant of *Streptococcus pyogenes*. J. Antimicrob. Chemother. 51, 707–710. 10.1093/jac/dkg121 12615875

[B17] Laponogov I. Veselkov D. A. Sohi M. K. Pan X. S. Achari A. Yang C.. (2007). Breakage-reunion domain of Streptococcus pneumoniae topoisomerase IV: crystal structure of a gram-positive quinolone target. PLoS ONE 2:e301. 10.1371/journal.pone.0000301 17375187 PMC1810434

[B18] Lau E. H. Nishiura H. Cowling B. J. Ip D. K. Wu J. T. (2012). Scarlet fever outbreak, Hong Kong, 2011. Emerging Infect. Dis. 18, 1700–1702. 10.3201/eid1810.120062 23017843 PMC3471616

[B19] Lin J. N. Chang L. L. Lai C. H. Huang Y. H. Chen W. F. Yang C. H.. (2015). High prevalence of fluoroquinolone-nonsusceptible *Streptococcus pyogenes emm*12 in Taiwan. Diagn. Microbiol. Infect. Dis. 83, 187–192. 10.1016/j.diagmicrobio.2015.06.018 26234479

[B20] Luk E. Y. Lo J. Y. Li A. Z. Lau M. C. Cheung T. K. Wong A. Y.. (2012). Scarlet fever epidemic, Hong Kong, 2011. Emerging Infect. Dis. 18, 1658–1661. 10.3201/eid1810.111900 23018120 PMC3471614

[B21] Malhotra-Kumar S. Lammens C. Chapelle S. Mallentjer C. Weyler J. Goossens H. (2005). Clonal spread of fluoroquinolone non-susceptible *Streptococcus pyogenes*. J. Antimicrob. Chemother. 55, 320–325. 10.1093/jac/dki011 15705642

[B22] Malhotra-Kumar S. Van Heirstraeten L. Lammens C. Chapelle S. Goossens H. (2009). Emergence of high-level fluoroquinolone resistance in *emm*6 *Streptococcus pyogenes* and in vitro resistance selection with ciprofloxacin, levofloxacin and moxifloxacin. J. Antimicrob. Chemother. 63, 886–894. 10.1093/jac/dkp057 19279051

[B23] Montes M. Tamayo E. Orden B. Larruskain J. Perez-Trallero E. (2010). Prevalence and clonal characterization of *Streptococcus pyogenes* clinical isolates with reduced fluoroquinolone susceptibility in Spain. Antimicrob. Agents Chemother. 54, 93–97. 10.1128/AAC.00780-09 19805559 PMC2798512

[B24] Orscheln R. C. Johnson D. R. Olson S. M. Presti R. M. Martin J. M. Kaplan E. L.. (2005). Intrinsic reduced susceptibility of serotype 6 *Streptococcus pyogenes* to fluoroquinolone antibiotics. J. Infect. Dis. 191, 1272–1279. 10.1086/428856 15776373

[B25] Pérez-Trallero E. Montes M. Orden B. Tamayo E. García-Arenzana J. M. Marimón J. M. (2007). Phenotypic and genotypic characterization of *Streptococcus pyogenes* isolates displaying the MLSB phenotype of macrolide resistance in Spain, 1999 to 2005. Antimicrob. Agents Chemother. 51, 1228–1233. 10.1128/AAC.01054-06 17242142 PMC1855467

[B26] Petrelli D. Di Luca M. C. Prenna M. Bernaschi P. Repetto A. Vitali L. A. (2014). Characterization of levofloxacin non-susceptible clinical *Streptococcus pyogenes* isolated in the central part of Italy. Eur. J. Clin. Microbiol. Infect. Dis. 33, 241–244. 10.1007/s10096-013-1950-5 24002218

[B27] Pinho M. D. Melo-Cristino J. Ramirez M. (2010). Fluoroquinolone resistance in *Streptococcus dysgalactiae* subsp. equisimilis and evidence for a shared global gene pool with *Streptococcus pyogenes*. Antimicrob. Agents Chemother. 54, 1769–1777. 10.1128/AAC.01377-09 20145082 PMC2863612

[B28] Pires R. Ardanuy C. Rolo D. Morais A. Brito-Avô A. Gonçalo-Marques J.. (2010). Emergence of ciprofloxacin-nonsusceptible *Streptococcus pyogenes* isolates from healthy children and pediatric patients in Portugal. Antimicrob. Agents Chemother. 54, 2677–2680. 10.1128/AAC.01536-09 20350943 PMC2876401

[B29] Pletz M. W. McGee L. Van Beneden C. A. Petit S. Bardsley M. Barlow M.. (2006). Fluoroquinolone resistance in invasive *Streptococcus pyogenes* isolates due to spontaneous mutation and horizontal gene transfer. Antimicrob. Agents Chemother. 50, 943–948. 10.1128/AAC.50.3.943-948.2006 16495255 PMC1426425

[B30] Powis J. McGeer A. Duncan C. Goren R. de Azavedo J. C. Bast D. J.. (2005). Prevalence and characterization of invasive isolates of *Streptococcus pyogenes* with reduced susceptibility to fluoroquinolones. Antimicrob. Agents Chemother. 49, 2130–2132. 10.1128/AAC.49.5.2130-2132.2005 15855546 PMC1087656

[B31] Reinert R. R. Lütticken R. Al-Lahham A. (2004). High-level fluoroquinolone resistance in a clinical *Streptoccoccus pyogenes* isolate in Germany. Clin. Microbiol. Infect. 10, 659–662. 10.1111/j.1469-0691.2004.00890.x 15214881

[B32] Richter S. S. Diekema D. J. Heilmann K. P. Almer L. S. Shortridge V. D. Zeitler R.. (2003). Fluoroquinolone resistance in *Streptococcus pyogenes*. Clin. Infect. Dis. 36, 380–383. 10.1086/345904 12539083

[B33] Rivera A. Rebollo M. Sánchez F. Navarro F. Miró E. Mirelis B.. (2005). Characterisation of fluoroquinolone-resistant clinical isolates of *Streptococcus pyogenes* in Barcelona, Spain. Clin. Microbiol. Infect. 11, 759–761. 10.1111/j.1469-0691.2005.01216.x 16104992

[B34] Smeesters P. R. Vergison A. Junior D. C. Van Melderen L. (2009). Emerging fluoroquinolone-non-susceptible group A Streptococci in two different paediatric populations. Int. J. Antimicrob. Agents 34, 44–49. 10.1016/j.ijantimicag.2009.01.012 19269141

[B35] Stahlmann R. Merker H. J. Hinz N. Chahoud I. Webb J. Heger W.. (1990). Ofloxacin in juvenile non-human primates and rats. Arthropathia and drug plasma concentrations. Arch. Toxicol. 64, 193–204. 10.1007/BF02010725 2115323

[B36] Tse H. Bao J. Y. Davies M. R. Maamary P. Tsoi H. W. Tong A. H.. (2012). Molecular characterization of the 2011 Hong Kong scarlet Fever outbreak. J. Infect. Dis. 206, 341–351. 10.1093/infdis/jis362 22615319 PMC4125623

[B37] Van Boeckel T. P. Gandra S. Ashok A. Caudron Q. Grenfell B. T. Levin S. A.. (2014). Global antibiotic consumption 2000 to 2010: an analysis of national pharmaceutical sales data. Lancet Infect. Dis. 14, 742–750. 10.1016/S1473-3099(14)70780-7 25022435

[B38] Van Heirstraeten L. Leten G. Lammens C. Goossens H. Malhotra-Kumar S. (2012). Increase in fluoroquinolone non-susceptibility among clinical *Streptococcus pyogenes* in Belgium during 2007-10. J. Antimicrob. Chemother. 67, 2602–2605. 10.1093/jac/dks281 22815354

[B39] Wajima T. Morozumi M. Chiba N. Shouji M. Iwata S. Sakata H.. (2013). Associations of macrolide and fluoroquinolone resistance with molecular typing in *Streptococcus pyogenes* from invasive infections, 2010-2012. Int. J. Antimicrob. Agents 42, 447–449. 10.1016/j.ijantimicag.2013.06.022 23988719

[B40] Wajima T. Murayama S. Y. Sunaoshi K. Nakayama E. Sunakawa K. Ubukata K. (2008). Distribution of *emm* type and antibiotic susceptibility of group A streptococci causing invasive and noninvasive disease. J. Med. Microbiol. 57(Pt 11), 1383–1388. 10.1099/jmm.0.2008/002642-0 18927416

[B41] Walker M. J. Barnett T. C. McArthur J. D. Cole J. N. Gillen C. M. Henningham A.. (2014). Disease manifestations and pathogenic mechanisms of Group A *Streptococcus*. Clin. Microbiol. Rev. 27, 264–301. 10.1128/CMR.00101-13 24696436 PMC3993104

[B42] Yan S. S. Fox M. L. Holland S. M. Stock F. Gill V. J. Fedorko D. P. (2000). Resistance to multiple fluoroquinolones in a clinical isolate of *Streptococcus pyogenes*: identification of *gyrA* and *parC* and specification of point mutations associated with resistance. Antimicrob. Agents Chemother. 44, 3196–3198. 10.1128/AAC.44.11.3196-3198.2000 11036052 PMC101632

[B43] Yan S. S. Schreckenberger P. C. Zheng X. Nelson N. A. Harrington S. M. Tjhio J.. (2008). An intrinsic pattern of reduced susceptibility to fluoroquinolones in pediatric isolates of *Streptococcus pyogenes*. Diagn. Microbiol. Infect. Dis. 62, 205–209. 10.1016/j.diagmicrobio.2008.04.018 18554840 PMC2572761

[B44] Yang P. Peng X. Zhang D. Wu S. Liu Y. Cui S.. (2013). Characteristics of group A *Streptococcus* strains circulating during scarlet fever epidemic, Beijing, China, 2011. Emerging Infect. Dis. 19, 909–915. 10.3201/eid1906.121020 23735582 PMC4816378

