# Supplementary material for: Identification and Characterization of Fluoroquinolone Non-susceptible Streptococcus pyogenes Clones Harboring Tetracycline and Macrolide Resistance in Shanghai, China
Source: Front Microbiol. 2018 Mar 23;9:542. doi: 10.3389/fmicb.2018.00542 (PMC5876283; doi:10.3389/fmicb.2018.00542)
Supplement: Supplementary file 1 [file Table1.DOCX]

**Table S1. Accession number of genome sequences of Streptococcus pyogenes isolates from Hong Kong and mainland China¶**

| Isolate | *emm* type | Accession No. | Year | Patient age (years) | District | Clinical presentation | Resistance ‡ | Resistance associated gene | | Resistance associated ParC mutation | *parC*  allele |
| --- | --- | --- | --- | --- | --- | --- | --- | --- | --- | --- | --- |
|  |  |  |  |  |  |  |  | *tetM* | *ermB* |  |  |
| HKU16 | 12 | AFRY01000001 | 2011 | 6 | Hong Kong | Scarlet fever | Ery/CLD/TET | + | + | - | ND |
| HKU17 | 12 | ERR060216 | 2011 | 35 | Hong Kong | Pharyngitis | Ery/CLD/TET | + | + | - | ND |
| HKU18 | 12 | ERR060217 | 2011 | 27 | Hong Kong | Flu | Ery/CLD/TET | + | + | - | ND |
| HKU19 | 12 | ERR060218 | 2011 | 13 | Hong Kong | Tonsillitis | Ery/CLD | - | - | - | ND |
| HKU20 | 12 | ERR060219 | 2011 | 4 | Hong Kong | Scarlet fever | Ery/CLD/TET | + | + | - | ND |
| HKU21 | 12 | ERR060220 | 2011 | 6 | Hong Kong | Scarlet fever | Ery/CLD/TET | + | + | - | ND |
| HKU22 | 12 | ERR060221 | 2011 | 4 | Hong Kong | ND† | CLD | - | - | - | ND |
| HKU23 | 12 | ERR060222 | 2011 | 5 | Hong Kong | Tonsillitis | Ery/CLD/TET | + | + | - | ND |
| HKU24 | 12 | ERR060223 | 2011 | 5 | Hong Kong | Fever | Ery/CLD/TET | + | + | - | ND |
| HKU25 | 12 | ERR060224 | 2011 | 5 | Hong Kong | Scarlet fever | Ery/CLD/TET | + | + | - | ND |
| HKU26 | 12 | ERR060225 | 2011 | 2 | Hong Kong | Pharyngitis | Ery/CLD/TET | + | + | - | ND |
| HKU27 | 12 | ERR060226 | 2011 | 39 | Hong Kong | Urinary tract infection | ND | - | - | - | ND |
| HKU28 | 12 | ERR060227 | 2011 | 9 | Hong Kong | Tonsillitis | Ery/CLD/TET | + | + | - | ND |
| HKU29 | 12 | ERR060228 | 2011 | 7 | Hong Kong | Tonsillitis | Ery/CLD/TET | + | + | - | ND |
| HKU30 | 12 | ERR054999 | 2011 | 4 | Hong Kong | Scarlet fever | Ery/CLD/TET | + | + | - | ND |
| HKU31 | 12 | ERR060229 | 2011 | 21 | Hong Kong | Vaginal discharge | ND | - | - | - | ND |
| HKU32 | 12 | ERR060230 | 2011 | 33 | Hong Kong | Sexual transmitted infection | Ery/CLD/TET | + | + | S79F | SHparC2 |
| HKU33 | 12 | ERR060231 | 2011 | 40 | Hong Kong | Cough | ND | - | - | - | ND |
| HKU34 | 12 | ERR060232 | 2011 | 61 | Hong Kong | Cough | Ery/CLD/TET | + | + | - | ND |
| HKU35 | 12 | ERR060233 | 2011 | 1 | Hong Kong | Eczema | Ery/CLD/TET | + | + | - | ND |
| HKU36 | 12 | ERR060234 | 2011 | 4 | Hong Kong | Urinary tract infection | Ery/CLD/TET | + | + | - | ND |
| HKU37 | 12 | ERR060235 | 2011 | 36 | Hong Kong | Bronchiolitis | Ery/CLD/TET | + | + | - | ND |
| HKU38 | 12 | ERR060236 | 2011 | 8 | Hong Kong | Vaginal discharge | Ery/CLD/TET | + | + | S79F | SHparC2 |
| HKU39 | 12 | ERR060237 | 2011 | 3.5 | Hong Kong | Pharyngitis | Ery/CLD/TET | + | + | - | ND |
| HKU40 | 12 | ERR060238 | 2011 | 9 | Hong Kong | Upper respiratory tract infection | Ery/CLD/TET | + | + | - | ND |
| HKU41 | 12 | ERR060239 | 2011 | 4 | Hong Kong | Possible strep throat | Ery/CLD/TET | + | + | - | ND |
| HKU42 | 12 | ERR060240 | 2011 | 8 | Hong Kong | Tonsillitis | Ery/CLD/TET | + | + | - | ND |
| HKU43 | 12 | ERR060241 | 2011 | 39 | Hong Kong | Tonsillitis | ND | - | - | - | ND |
| HKU44 | 12 | ERR060242 | 2011 | 37 | Hong Kong | Tonsillitis | Ery/CLD/TET | + | + | - | ND |
| HKU45 | 12 | ERR060243 | 2011 | 9 | Hong Kong | Pharyngotonsillitis | ND | - | - | - | ND |
| HKU46 | 12 | ERR060244 | 2011 | 71 | Hong Kong | Infected uterine pessary | Ery/CLD/TET | + | + | - | ND |
| HKU47 | 12 | ERR060245 | 2011 | 48 | Hong Kong | Pharyngitis | Ery/CLD/TET | + | + | - | ND |
| HKU48 | 12 | ERR060246 | 2011 | 40 | Hong Kong | Pharyngitis | Ery/CLD/TET | + | + | - | ND |
| HKU49 | 12 | ERR060247 | 2011 | 10 | Hong Kong | Urinary tract infection | ND | - | - | S79F | SHparC2 |
| HKU50 | 12 | ERR060248 | 2011 | 8 | Hong Kong | Tonsillitis | ND | - | - | - | ND |
| HKU51 | 12 | ERR060249 | 2011 | 48 | Hong Kong | Cough | Ery/CLD/TET | + | + | S79F | SHparC2 |
| HKU52 | 12 | ERR060250 | 2011 | 31 | Hong Kong | Pharyngitis | ND | - | - | S79F | SHparC2 |
| HKU53 | 12 | ERR060251 | 2011 | 8 | Hong Kong | Tonsillitis | Ery/CLD/TET | + | + | - | ND |
| HKU54 | 12 | ERR060252 | 2011 | 33 | Hong Kong | PV discharge | Ery/CLD/TET | + | + | - | ND |
| HKU55 | 12 | ERR060253 | 2011 | 16 | Hong Kong | Fever | ND | - | - | - | ND |
| HKU56 | 12 | ERR060254 | 2011 | 16 | Hong Kong | Fever | ND | - | - | - | ND |
| HKU58 | 12 | ERR060256 | 2011 | 47 | Hong Kong | Upper respiratory illness | Ery/CLD/TET | + | + | - | ND |
| HKU74 | 12 | ERR060257 | 2005 | 6 | Hong Kong | Acute tonsilitis | ND | - | - | - | ND |
| HKU81 | 12 | ERR060258 | 2005 | 5 | Hong Kong | Rash & congested throat | ND | - | - | - | ND |
| HKU84 | 12 | ERR060259 | 2005 | 3 | Hong Kong | Acute Tonsilitis | Ery/CLD/TET | + | + | - | ND |
| HKU86 | 12 | ERR060260 | 2005 | 4 | Hong Kong | Pharyngitis | Ery/CLD/TET | + | + | - | ND |
| HKU98 | 12 | ERR060262 | 2006 | 4 | Hong Kong | Scarlet fever | Ery/CLD/TET | + | + | - | ND |
| HKU99 | 12 | ERR060263 | 2006 | 3 | Hong Kong | Acute tonsilitis | ND | - | - | - | ND |
| HKU104 | 12 | ERR060264 | 2007 | 39 | Hong Kong | Sepsis | ND | - | - | - | ND |
| HKU111 | 12 | ERR060265 | 2007 | 5 | Hong Kong | Scarlet fever | Ery/CLD/TET | + | + | - | ND |
| HKU116 | 12 | ERR060266 | 2007 | 8 | Hong Kong | Acute tonsilitis | Ery/CLD/TET | + | + | - | ND |
| HKU127 | 12 | ERR060267 | 2008 | 30 | Hong Kong | Wound infection (burn) | ND | - | - | - | ND |
| HKU137 | 12 | ERR060268 | 2009 | 6 | Hong Kong | Acute tonsilitis | ND | - | - | - | ND |
| HKU138 | 12 | ERR060269 | 2009 | 12 | Hong Kong | Acute tonsilitis | Ery/CLD/TET | + | + | - | ND |
| HKU139 | 12 | ERR060270 | 2009 | 5 | Hong Kong | Scarlet fever | ND | - | - | - | ND |
| HKU151 | 12 | ERR060271 | 2010 | 3 | Hong Kong | Scarlet fever | ND | - | - | - | ND |
| HKU152 | 12 | ERR060272 | 2010 | 6 | Hong Kong | Pharyngitis | ND | - | - | - | ND |
| HKU153 | 12 | ERR060273 | 2010 | 5 | Hong Kong | Scarlet fever | Ery/CLD/TET | + | + | - | ND |
| HKU157 | 12 | ERR060274 | 2010 | 5 | Hong Kong | Infection & rash | Ery/CLD/TET | + | + | - | ND |
| HKU160 | 12 | ERR060275 | 2010 | 4 | Hong Kong | Bil otitis media & rash & tonsilopharyngitis | ND | - | - | S79F | SHparC2 |
| HKU161 | 12 | ERR060276 | 2010 | 5 | Hong Kong | Henoch Schonlein purpura | Ery/CLD/TET | + | + | - | ND |
| HKU165 | 12 | ERR060277 | 2005 | 56 | Hong Kong | Shortness of breath & asthma | Ery/CLD/TET | + | - | - | ND |
| HKU189 | 12 | ERR060279 | 2005 | 2 | Hong Kong | Pharyngitis | Ery/CLD/TET | + | + | - | ND |
| HKU283 | 12 | ERR060282 | 2008 | 4 | Hong Kong | Pharyngitis | Ery/CLD/TET | + | + | - | ND |
| HKU288 | 12 | ERR060283 | 2008 | 6 | Hong Kong | Acute tonsilitis | Ery/CLD/TET | + | + | S79F | SHparC2 |
| HKU294 | 12 | ERR060284 | 2008 | 7 | Hong Kong | Upper respiratory tract infection & sore throat | Ery/CLD/TET | + | + | S79F | SHparC2 |
| HKU306 | 12 | ERR060285 | 2011 | 6 | Hong Kong | Scarlet fever | Ery/CLD/TET | + | + | - | ND |
| HKU309 | 12 | ERR060286 | 2011 | 5 | Hong Kong | Viral rash | Ery/CLD/TET | + | + | - | ND |
| HKU311 | 12 | ERR060287 | 2011 | 7 | Hong Kong | Scarlet fever | Ery/CLD/TET | + | + | - | ND |
| HKU315 | 12 | ERR060288 | 2011 | 4 | Hong Kong | Scarlet fever | Ery/CLD/TET | + | + | - | ND |
| HKU320 | 12 | ERR060289 | 2011 | 4 | Hong Kong | Vulval pain & dysuria | Ery/CLD/TET | + | + | - | ND |
| HKU322 | 12 | ERR060290 | 2011 | 4 | Hong Kong | Scarlet fever | Ery/CLD/TET | + | + | - | ND |
| HKU324 | 12 | ERR060291 | 2011 | 2 | Hong Kong | Acute tonsilitis | Ery/CLD/TET | + | + | - | ND |
| HKU326 | 12 | ERR060292 | 2011 | 32 | Hong Kong | Acute tonsilitis | Ery/CLD/TET | + | + | - | ND |
| HKU327 | 12 | ERR060293 | 2011 | 9 | Hong Kong | Scarlet fever | Ery/CLD/TET | + | + | - | ND |
| HKU328 | 12 | ERR060294 | 2011 | 32 | Hong Kong | Acute tonsilitis | Ery/CLD/TET | + | + | - | ND |
| HKU330 | 12 | ERR060295 | 2011 | 8 | Hong Kong | Otitis externa & facial cellulitis | Ery/CLD/TET | + | + | - | ND |
| HKU331 | 12 | ERR060296 | 2011 | 7 | Hong Kong | Scarlet fever | Ery/CLD/TET | + | + | - | ND |
| HKU333 | 12 | ERR060297 | 2011 | 1 | Hong Kong | Scarlet fever | Ery/CLD/TET | + | + | - | ND |
| HKU334 | 12 | ERR060298 | 2011 | 8 | Hong Kong | Otitis externa & facial cellulitis | Ery/CLD/TET | + | + | - | ND |
| HKU335 | 12 | ERR060299 | 2011 | 6 | Hong Kong | Scarlet fever | Ery/CLD/TET | + | + | - | ND |
| HKU336 | 12 | ERR060300 | 2011 | 8 | Hong Kong | Scarlet fever | Ery/CLD/TET | + | + | - | ND |
| HKU343 | 12 | ERR060301 | 2011 | 8 | Hong Kong | Scarlet fever | Ery/CLD/TET | + | + | - | ND |
| HKU347 | 12 | ERR060302 | 2011 | 4 | Hong Kong | Pharyngitis | Ery/CLD/TET | + | + | - | ND |
| HKU349 | 12 | ERR060303 | 2011 | 5 | Hong Kong | Left thumb blister | Ery/CLD/TET | + | + | - | ND |
| HKU354 | 12 | ERR060304 | 2011 | 5 | Hong Kong | Pharyngitis | Ery/CLD/TET | + | + | - | ND |
| HKU355 | 12 | ERR060305 | 2011 | 14 | Hong Kong | Scarlet fever | Ery/CLD/TET | + | + | - | ND |
| HKU356 | 12 | ERR060306 | 2011 | 11 | Hong Kong | Scarlet fever | Ery/CLD/TET | + | + | - | ND |
| HKU357 | 12 | ERR060307 | 2011 | 9 | Hong Kong | Pharyngitis | Ery/CLD/TET | + | + | - | ND |
| HKU358 | 12 | ERR060308 | 2011 | 8 | Hong Kong | Scarlet fever | Ery/CLD/TET | + | + | - | ND |
| HKU359 | 12 | ERR060309 | 2011 | 8 | Hong Kong | Scarlet fever | Ery/CLD/TET | + | + | - | ND |
| HKU360 | 12 | ERR060310 | 2011 | 7 | Hong Kong | Scarlet fever | Ery/CLD/TET | + | + | - | ND |
| HKU361 | 12 | ERR060311 | 2008 | 4 | Hong Kong | Pharyngitis | Ery/CLD/TET | + | + | - | ND |
| HKU362 | 12 | ERR060312 | 2008 | 50 | Hong Kong | Acute pharyngitis | ND | - | - | - | ND |
| HKU363 | 12 | ERR060313 | 2008 | 79 | Hong Kong | Surgical wound infection | ND | - | - | - | ND |
| HKU364 | 12 | ERR060314 | 2008 | 34 | Hong Kong | Vaginal discharge | ND | - | - | - | ND |
| HKU366 | 12 | ERR060316 | 2009 | 35 | Hong Kong | Acute tonsillitis | ND | - | - | - | ND |
| HKU367 | 12 | ERR060317 | 2010 | 29 | Hong Kong | Vaginal discharge | ND | - | - | - | ND |
| HKU368 | 12 | ERR060318 | 2010 | 15 | Hong Kong | Vaginal discharge | ND | - | - | - | ND |
| HKU369 | 12 | ERR060319 | 2010 | 41 | Hong Kong | Tonsillitis | ND | - | - | - | ND |
| HKU370 | 12 | ERR060320 | 2010 | 5 | Hong Kong | Acute pharyngitis | ND | - | - | - | ND |
| HKU371 | 12 | ERR060321 | 2010 | 29 | Hong Kong | Acute tonsillitis | ND | - | - | - | ND |
| HKU372 | 12 | ERR060322 | 2010 | 7 | Hong Kong | Vaginal discharge | Ery/CLD | - | + | - | ND |
| HKU373 | 12 | ERR060323 | 2011 | 6 | Hong Kong | Scarlet fever | Ery/CLD/TET | + | + | S79F | SHparC2 |
| HKU374 | 12 | ERR060324 | 2011 | 3 | Hong Kong | Scarlet fever | Ery/CLD/TET | + | + | - | ND |
| HKU375 | 12 | ERR060325 | 2011 | 6 | Hong Kong | Scarlet fever | Ery/CLD/TET | + | + | S79F | SHparC2 |
| HKU376 | 12 | ERR060326 | 2011 | 5 | Hong Kong | Scarlet fever | Ery/CLD/TET | + | + | S79F | SHparC2 |
| HKU377 | 12 | ERR060327 | 2011 | 5 | Hong Kong | Scarlet fever | Ery/CLD/TET | + | + | - | ND |
| HKU378 | 12 | ERR060328 | 2011 | 8 | Hong Kong | Scarlet fever | Ery/CLD/TET | + | + | - | ND |
| HKU379 | 12 | ERR060329 | 2011 | 8 | Hong Kong | Scarlet fever | Ery/CLD/TET | + | + | - | ND |
| HKU380 | 12 | ERR060330 | 2011 | 4 | Hong Kong | Scarlet fever | Ery/CLD/TET | + | + | - | ND |
| HKU381 | 12 | ERR060331 | 2011 | 5 | Hong Kong | Scarlet fever | ND | - | - | - | ND |
| HKU382 | 12 | ERR060332 | 2011 | 4 | Hong Kong | Scarlet fever | ND | - | - | S79F | SHparC2 |
| HKU383 | 12 | ERR060333 | 2011 | 3 | Hong Kong | Scarlet fever | Ery/CLD/TET | + | + | - | ND |
| HKU384 | 12 | ERR060334 | 2011 | 6 | Hong Kong | Scarlet fever | Ery/CLD/TET | + | + | - | ND |
| HKU385 | 12 | ERR060335 | 2011 | 6 | Hong Kong | Scarlet fever | Ery/CLD/TET | + | + | - | ND |
| HKU386 | 12 | ERR060336 | 2011 | 4 | Hong Kong | Scarlet fever | Ery/CLD/TET | + | + | - | ND |
| HKU387 | 12 | ERR060337 | 2011 | 3 | Hong Kong | Scarlet fever | Ery/CLD/TET | + | + | - | ND |
| HKU388 | 12 | ERR060338 | 2011 | 6 | Hong Kong | Scarlet fever | ND | - | - | - | ND |
| HKU389 | 12 | ERR060339 | 2011 | 6 | Hong Kong | Scarlet fever | ND | - | - | - | ND |
| HKU390 | 12 | ERR060340 | 2011 | 3 | Hong Kong | Scarlet fever | Ery/CLD/TET | + | + | - | ND |
| HKU391 | 12 | ERR060341 | 2011 | 5 | Hong Kong | Scarlet fever | Ery/CLD/TET | + | + | - | ND |
| HKU392 | 12 | ERR060342 | 2011 | 3 | Hong Kong | Scarlet fever | Ery/CLD/TET | + | + | - | ND |
| HKU393 | 12 | ERR060343 | 2011 | 5 | Hong Kong | Scarlet fever | Ery/CLD/TET | + | + | - | ND |
| HKU394 | 12 | ERR060344 | 2011 | 4 | Hong Kong | Scarlet fever | Ery/CLD/TET | + | + | - | ND |
| HKU395 | 12 | ERR060345 | 2011 | 6 | Hong Kong | Scarlet fever | Ery/CLD/TET | + | + | - | ND |
| HKU396 | 12 | ERR060346 | 2011 | 10 | Hong Kong | Scarlet fever | Ery/CLD/TET | + | + | - | ND |
| HKU397 | 12 | ERR060347 | 2011 | 14 | Hong Kong | Scarlet fever | Ery/CLD/TET | + | + | - | ND |
| HKU398 | 12 | ERR060348 | 2011 | 4 | Hong Kong | Scarlet fever | Ery/CLD/TET | + | + | - | ND |
| HKU399 | 12 | ERR060349 | 2011 | 6 | Hong Kong | Scarlet fever | Ery/CLD/TET | + | + | - | ND |
| HKU400 | 12 | ERR060350 | 2011 | 6 | Hong Kong | Scarlet fever | Ery/CLD/TET | + | + | - | ND |
| HKU401 | 12 | ERR060351 | 2011 | 8 | Hong Kong | Scarlet fever | ND | - | - | - | ND |
| HKU402 | 12 | ERR060352 | 2011 | 6 | Hong Kong | Scarlet fever | Ery/CLD/TET | + | + | - | ND |
| HKU403 | 12 | ERR060353 | 2011 | 2 | Hong Kong | Scarlet fever | Ery/CLD/TET | + | + | - | ND |
| HLJGAS12011 | 12 | ALKE00000000 | 2011 | <12 | mainland China | Scarlet fever | ND | + | + | - | ND |
| BJCYGAS15 | 12 | ALKD00000000 | 2011 | <12 | mainland China | Scarlet fever | ND | + | + | - | ND |
| BJCYGAS112 | 1 | ERR986048 | 2011 | 3 | mainland China | Scarlet fever | Ery/CLD/TET | + | + | - | ND |
| BJCYGAS184 | 1 | ERR986049 | 2011 | 11 | mainland China | Scarlet fever | Ery/CLD/TET | + | + | - | ND |
| BJCYGAS52 | 1 | ERR986047 | 2011 | 13 | mainland China | Pharyngitis | Ery/CLD/TET | + | + | - | ND |
| BJGAS0403 | 1 | ERR986050 | 2004 | <15 | mainland China | Scarlet fever | ND | + | + | - | ND |
| BJGAS0501 | 1 | ERR986051 | 2005 | <15 | mainland China | Scarlet fever | ND | + | + | - | ND |
| BJGAS0601 | 1 | ERR986052 | 2006 | <15 | mainland China | Scarlet fever | ND | + | + | - | ND |
| BJGAS0602 | 1 | ERR986053 | 2006 | <15 | mainland China | Scarlet fever | ND | + | + | - | ND |
| BJGAS0701 | 1 | ERR986054 | 2007 | <15 | mainland China | Scarlet fever | ND | + | + | - | ND |
| BJGAS1001 | 1 | ERR986055 | 2010 | <15 | mainland China | Scarlet fever | ND | + | + | - | ND |
| BJXCGAS02 | 1 | ERR986056 | 2011 | 9 | mainland China | Scarlet fever | ND | + | + | - | ND |
| BJXCGAS05 | 1 | ERR986057 | 2011 | 4 | mainland China | Scarlet fever | ND | + | + | - | ND |
| BJYCGAS-0801 | 1 | ERR986058 | 2008 | <15 | mainland China | Scarlet fever | ND | + | + | - | ND |
| HKU416 | 1 | ERR172161 | 2012 | 14 | Hong Kong | Scarlet fever | Ery/CLD/TET | + | + | - | ND |
| HKU417 | 1 | ERR172162 | 2012 | 11 | Hong Kong | Scarlet fever | Ery/CLD/TET | + | + | - | ND |
| HKU419 | 1 | ERR172163 | 2012 | 8 | Hong Kong | Scarlet fever | Ery/CLD/TET | + | + | - | ND |
| HKU421 | 1 | ERR172164 | 2012 | 3 | Hong Kong | Rash | Ery/CLD/TET | + | + | - | ND |
| HKU425 | 1 | ERR172165 | 2012 | 48 | Hong Kong | Necrotizing fasciitis | ND | - | - | - | ND |
| HKU434 | 1 | ERR172166 | 2011 | 6 | Hong Kong | Scarlet fever | ND | - | - | - | ND |
| HKU444 | 1 | ERR172167 | 2011 | 6 | Hong Kong | Scarlet fever | Ery/CLD/TET | + | + | - | ND |
| HKU463 | 1 | ERR172168 | 2012 | 5 | Hong Kong | Upper respiratory tract infection | Ery/CLD/TET | + | + | - | ND |
| HKU464 | 1 | ERR172169 | 2012 | 76 | Hong Kong | Pneumonia | Ery/CLD/TET | + | + | - | ND |
| HKU471 | 1 | ERR172170 | 2012 | 4 | Hong Kong | Scarlet fever | ND | - | - | - | ND |
| HKU474 | 1 | ERR172171 | 2012 | 12 | Hong Kong | Scarlet fever | ND | - | - | - | ND |
| HKU480 | 1 | ERR172172 | 2012 | 8 | Hong Kong | Scarlet fever, necrotizing pneumonia | Ery/CLD/TET | + | + | - | ND |
| HKU484 | 1 | ERR172173 | 2012 | 7 | Hong Kong | Scarlet fever | ND | - | - | - | ND |
| HKU485 | 1 | ERR172174 | 2012 | 63 | Hong Kong | Fever, left chronic suppurative otitis media | ND | - | - | - | ND |
| HKU486 | 1 | ERR172175 | 2011 | 6 | Hong Kong | Fever | Ery/CLD/TET | + | + | - | ND |
| HKU487 | 1 | ERR172176 | 2011 | 10 | Hong Kong | Scarlet fever | Ery/CLD/TET | + | + | - | ND |
| HKU488 | 1 | ERR172177 | 2012 | 8 | Hong Kong | Scarlet fever | Ery/CLD/TET | + | + | - | ND |
| HKU489 | 1 | ERR172178 | 2012 | 10 | Hong Kong | Henoch-Schönlein purpura | Ery/CLD/TET | + | + | - | ND |
| HLJGAS2022 | 1 | ERR986059 | 2011 | 7 | mainland China | Scarlet fever | Ery/CLD/TET | + | + | D83G | SHparC10 |
| SYGAS06 | 1 | ERR986060 | 2011 | 11 | mainland China | ND | ND | + | + | - | ND |
| TJ11-007 | 1 | ERR986061 | 2011 | 4 | mainland China | Scarlet fever | Ery/CLD/TET | + | + | - | ND |
| TJ11-008 | 1 | ERR986062 | 2011 | 9 | mainland China | Scarlet fever | Ery/CLD/TET | + | + | - | ND |

¶ Except data of ParC mutation and *parC* allele are from this study, other data is from references (Davies et al. 2015) and (Ben Zakour *et al*. 2015).

‡ Ery, erythromycin; CLD, clindamycin; TET, tetracycline.

† ND, not determined.

**Table S2 Accession number of *parC* quinolone resistance-determining region (QRDR) sequences from different streptococcus species**

| Isolate | Accession number | Fluoroquinolone susceptibility* | Country | Species |
| --- | --- | --- | --- | --- |
| SF370 | AE004092 | S | USA | *S. pyogenes* |
| NCTC 8181T | AB101464 | S | Japan | *S. agalactiae* |
| GTC1966 | AB101465 | R | Japan | *S. agalactiae* |
| GTC1967 | AB101466 | R | Japan | *S. agalactiae* |
| GTC2001 | AB101467 | R | Japan | *S. agalactiae* |
| GIFU10482 | AB101468 | S | Japan | *S. agalactiae* |
| GIFU10483 | AB101469 | S | Japan | *S. agalactiae* |
| GIFU10484 | AB101470 | S | Japan | *S. agalactiae* |
| GTC431T | AB101472 | S | Japan | *S. dysgalactiae* subsp*. dysgalactiae* |
| GTC842 | AB101473 | S | Japan | *S. dysgalactiae* subsp*. equisimilis* |
| GTC423T | AB101474 | S | Japan | *S. canis* |
| GTC244T | AB101475 | S | Japan | *S. iniae* |
| GTC543T | AB101476 | S | Japan | *S. porcinus* |
| GTC269T | AB101477 | S | Japan | *S. equi* subsp*. equi* |
| GTC542T | AB101478 | S | Japan | *S. equi* subsp*. zooepidemicus* |
| GTC730T | AB101479 | S | Japan | *S. difficilis* |
| R6 | AE008482 | S | Japan | *S. pneumoniae* |
| SR27 | AF503576 | R | Japan | *S. pneumoniae* |
| 618280 | GU002027 | S | Portugal | *S. dysgalactiae* subsp. *equisimilis* |
| 223754 | GU002028 | S | Portugal | *S. dysgalactiae* subsp*. equisimilis* |
| 433437 | GU002029 | R | Portugal | *S. dysgalactiae* subsp*. equisimilis* |
| 544633 | GU002030 | R | Portugal | *S. dysgalactiae* subsp*. equisimilis* |
| 171712 | GU002031 | S | Portugal | *S. dysgalactiae* subsp*. equisimilis* |
| 529653 | GU002032 | R | Portugal | *S. dysgalactiae* subsp*. equisimilis* |
| 267366 | GU002033 | S | Portugal | *S. dysgalactiae* subsp*. equisimilis* |
| 194884 | GU002034 | R | Portugal | *S. dysgalactiae* subsp*. equisimilis* |
| 516887 | GU002035 | R | Portugal | *S. dysgalactiae* subsp*. equisimilis* |
| SH0344 | GU002036 | S | Portugal | *S. dysgalactiae* subsp*. equisimilis* |
| SH0330 | GU002037 | S | Portugal | *S. dysgalactiae* subsp*. equisimilis* |
| SH0102 | GU002038 | S | Portugal | *S. dysgalactiae* subsp*. equisimilis* |
| SH0972 | GU002039 | R | Portugal | *S. dysgalactiae* subsp*. equisimilis* |
| 460880 | GU002040 | S | Portugal | *S. dysgalactiae* subsp*. equisimilis* |
| 273600 | GU002041 | R | Portugal | *S. dysgalactiae* subsp*. equisimilis* |
| 313247 | GU002042 | S | Portugal | *S. dysgalactiae* subsp*. equisimilis* |
| 347460 | GU002043 | R | Portugal | *S. dysgalactiae* subsp*. equisimilis* |
| 423738 | GU002044 | S | Portugal | *S. dysgalactiae* subsp*. equisimilis* |
| 263149 | GU002045 | R | Portugal | *S. dysgalactiae* subsp*. equisimilis* |

* R, resistant; S, susceptible.

**References:**

Davies, M.R., Holden, M.T., Coupland, P., Chen, J.H., Venturini, C., Barnett, T.C., et al. (2015). Emergence of scarlet fever *Streptococcus* *pyogenes* *emm*12 clones in Hong Kong is associated with toxin acquisition and multidrug resistance. *Nat* *Genet* 47(1), 84-87. doi: 10.1038/ng.3147.

Ben Zakour, N.L., Davies, M.R., You, Y., Chen, J.H., Forde, B.M., Stanton-Cook, M., et al. (2015). Transfer of scarlet fever-associated elements into the group A *Streptococcus* M1T1 clone. *Sci Rep* 5, 15877. doi: 10.1038/srep15877.
